# 3R-Refinement principles: elevating rodent well-being and research quality

**DOI:** 10.1186/s42826-024-00198-3

**Published:** 2024-03-29

**Authors:** Puneet Rinwa, Marie Eriksson, Ian Cotgreave, Matilda Bäckberg

**Affiliations:** Department of Chemical and Pharmaceutical Safety, Division of Bioeconomy and Health, RISE Research Institutes of Sweden, Forskargatan 18, Södertälje, 151 36 Sweden

**Keywords:** 3R-Refinement principles, Scientific validity, Animal welfare, Research quality, Ethical considerations

## Abstract

This review article delves into the details of the 3R-Refinement principles as a vital framework for ethically sound rodent research laboratory. It highlights the core objective of the refinement protocol, namely, to enhance the well-being of laboratory animals while simultaneously improving the scientific validity of research outcomes. Through an exploration of key components of the refinement principles, the article outlines how these ethics should be implemented at various stages of animal experiments. It emphasizes the significance of enriched housing environments that reduce stress and encourage natural behaviors, non-restraint methods in handling and training, refined dosing and sampling techniques that prioritize animal comfort, the critical role of optimal pain management and the importance of regular animal welfare assessment in maintaining the rodents well-being. Additionally, the advantages of collaboration with animal care and ethics committees are also mentioned. The other half of the article explains the extensive benefits of the 3R-Refinement protocol such as heightened animal welfare, enhanced research quality, reduced variability, and positive feedback from researchers and animal care staff. Furthermore, it addresses avenues for promoting the adoption of the protocol, such as disseminating best practices, conducting training programs, and engaging with regulatory bodies. Overall, this article highlights the significance of 3R-Refinement protocol in aligning scientific advancement with ethical considerations along with shaping a more compassionate and responsible future for animal research.

## Background

Animal research has been pivotal in advancing scientific knowledge and medical breakthroughs, contributing significantly to our understanding of complex biological processes and human diseases [1]. However, with the privilege of conducting research on animals comes a profound responsibility to ensure the ethical treatment and welfare of the animals involved. In recognition of this responsibility, the 3R principles have emerged as a fundamental framework to guide researchers in conducting animal experiments ethically within research institutes. The 3R principles, introduced by Russell and Burch in 1959, advocate for the Replacement, Reduction, and Refinement of animal use in research [2]. While Replacement and Reduction focus on exploring alternative methods and minimizing the number of animals used, Refinement pertains to techniques that alleviate pain, distress, and suffering experienced by animals during experimentation [3]. By emphasizing the 3R-Refinement principles, researchers can seek to enhance animal welfare without compromising scientific integrity, resulting in more humane and reliable research outcomes.

The central objective of the refinement protocol is to strike a delicate balance between elevating the welfare of laboratory animals and refining the rigor of scientific investigations. Rats and mice, due to their genetic similarities with humans, rapid reproduction, cost-effectiveness, and ease of handling, are fundamental in scientific research and favorable for 3R-Refinement considerations. This review article, therefore, primarily focuses on these species, exploring refinement methods tailored to their unique characteristics. By carefully examining the key components of the refinement principles, this article reveals a comprehensive outline for their implementation across various stages of animal experiments. It focuses on creating enriched housing environments that mitigate stress and promote natural behaviors, adopting non-restraint methods for handling and training to foster trust and cooperation, and employing refined dosing and sampling techniques that prioritize the comfort of the animals [4, 5]. Apart from these, pain management and euthanasia techniques are crucial in maintaining animal welfare and ethical standards [6, 7]. To ensure continual animal welfare, regular monitoring and assessment are essential to gauge the well-being of the animals during the experiment. Collaboration with animal ethics committees fosters ethical research culture, while adhering to guidelines and regulations ensures responsible, transparent research practices. By embracing the 3R-Refinement principles, researchers can significantly impact animal welfare, improve the scientific validity and reliability of their findings, and reduce variability and the number of animals used [8]. In promoting the widespread adoption of Refinement practices, this article explores the value of collaboration with other research laboratories, sponsors and stakeholders, the dissemination of best practices and guidelines, the implementation of training programs and workshops, and the role of regulatory bodies and ethical review committees [9].

Overall, implementing the 3R-Refinement principles in rodent experiments is not only ethically crucial but also essential for enhancing the credibility and impact of scientific research. By adhering to these principles, researchers can strike a harmonious balance between scientific progress and compassionate treatment of laboratory rodents, ultimately advancing knowledge and bettering animal welfare in research institutes.

## Main text

### Key components of 3R-refinement principles

The 3R-Refinement principles constitute a pivotal framework in the pursuit of ethical and responsible laboratory animal research. The following key components of this protocol encompass various aspects that prioritize the welfare of laboratory animals while upholding scientific rigor.

#### Arrival and acclimatization of animals

The arrival of animals at a research facility marks the initial step in their journey within the experimental setting, and a well-structured protocol for unpacking animals is paramount to their welfare. A crucial aspect of unpacking involves ensuring proper disinfection to prevent cross-contamination between shipments of animals with varying health statuses. To address this, Loew (1980) suggests scheduling shipments from different sources to arrive on separate days [10]. Additionally, any abnormal findings with the animals should be promptly reported to the supplier or institution, and veterinarians at the receiving institute should conduct frequent observations within the first 24 to 48 h of receipt [11]. Establishing health monitoring procedures during quarantine for newly received animals is crucial to ensure their compatibility with institutional requirements. Quarantine duration and monitoring intensity may vary based on the animals’ source and its reliability [10].

Proper acclimatization mitigates stress and allows animals to adapt to their new environment, ensuring the reliability and ethicality of subsequent experiments [10]. It involves careful considerations, such as providing a conducive housing environment, tailored nutrition, and allowing for social interactions when applicable. Gradual acclimation to new environmental variables, such as food and water consumption, is essential; for instance, it has been documented that these factors are impacted during transfer to a different facility and typically take 3 to 5 days to normalize [12]. According to Gordon (2004), variations in cage construction and bedding type influence the maintenance of body temperature in mice as the animals consistently adapt to changes in the ambient environment [13]. Steady acclimatization after stressful transportation allows animals to familiarize themselves with the vivarium’s sounds, smells, and the presence of caretakers, all contributing to reduced stress levels [14]. Animals should be afforded a sense of safety when approaching animal technicians, while technicians should possess the ability to interpret animal cues and attentively observe their responses during interactions. Avishai-Eliner et al. (2002) demonstrates that regularly handling research animals during their early life, and particularly habituating them to a gentle treatment during this period, leads to decreased handling stress in later stages [15]. By adopting gentle handling and providing a calm environment, researchers can establish a positive rapport with the animals, setting the tone for humane and ethical treatment throughout their stay in the research institute.

#### Housing and enrichment environment

**Fig. 1:**
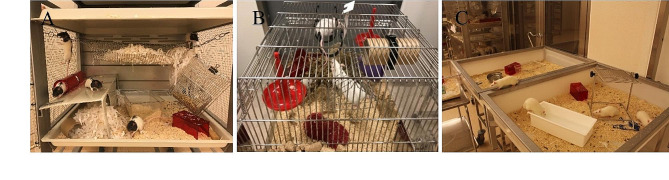
Housing, enrichment and open environment for rodents. (**A**) Larger cage space allows animals to engage in natural behaviors such as exploring, climbing, and nesting, which are important for their well-being and provides a more accurate representation of their natural behavior in a laboratory setting. These activities help reduce stress levels by providing mental stimulation and opportunities for physical exercise. (**B**) Providing rodents with toys to play and chew, places to hide, and objects to climb on offers mental stimulation. Just like humans, rodents benefit from engaging activities that keep their minds active and curious. This enrichment helps prevent boredom and promotes mental well-being. Enriched environments also improve the validity of research outcomes by reducing stress-related confounding factors. (**C**) In an observation box with open space, animals have the freedom to behave as they would in their natural habitat. Furthermore, having animals in an observation box with open space makes it easier for researchers to monitor them for any signs of clinical distress following treatment. With ample room to move and interact, researchers can closely observe the animals’ behavior, body language, and overall performance. This enhanced visibility enables early detection of any potential adverse effects of treatments or interventions, allowing prompt intervention and mitigation of discomfort or distress

Enriched housing environment not only promotes animal welfare but also has potential implications for research outcomes. Use of large cage spaces (e.g., repurposing rabbit cages for housing rats and rat cages for mice) allow rodents to engage in natural behaviors, including exploration, climbing, nesting, and burrowing, thus reducing stress, and promoting a more naturalistic living environment [16, 17]. Furthermore, the benefits of social housing for rodents cannot be understated. Group-housed rodents are more likely to experience positive social interactions, which can lead to reduced aggression and improved social skills [18]. However, it is essential to consider individual preferences and behavioral dynamics after forming and observing the social groups, as the compatibility of cage mates can significantly impact their well-being [19]. Incorporating a variety of enrichment materials, such as chewable toys, nesting material, hiding places, climbing/balancing challenges (e.g., trapeze swing, running wheels, ladders), digging possibilities, search for food options, social contacts etc. provide rodents with opportunities for mental stimulation and physical exercise [20, 21] (Fig. 1). These enrichments help prevent stereotypic behaviors that may arise from monotonous housing conditions, ultimately improving the animals’ emotional resilience and cognitive abilities [22]. While providing enrichment, it is crucial to regularly assess and rotate these items to maintain their novelty and effectiveness. Observation boxes create a semblance of open space for animals within a controlled environment allowing them to experience a more enriched setting, enhancing their overall well-being. They also serve as a valuable space for studying animals’ behavior and interactions. Researchers can closely analyze animal’s responses to different stimuli and monitor the effects of previous interactions. Thus, acknowledging the significance of providing stimulating and supportive living conditions for rodents fosters a friendly balance between ethical responsibilities and scientific advancement in research settings.

In certain situations, experiments may require smaller cages due to specific research needs or spatial constraints, which could limit the consistent application of enriched environments. This potential incompatibility between the ideal and the practical aspects of rodent housing across all experimental conditions underscores the importance of a subtle approach. This approach recognizes the varied demands of experiments while striving for optimal animal welfare within existing constraints. Benefiel et al. (2005) clearly advocated the improved animal welfare but also suggested that environmental enrichment has the potential for causing an increase in experimental variability and research outcomes [23]. Toth et al. (2011) highlighted that enriched environments should be carefully designed as to leave experimental results unaltered and recommended the evaluation of enrichment on an experiment-to-experiment basis [24]. Enrichment strategies, such as large and open cage environments, are particularly beneficial for experiments focused on toxicology, toxicity assessment, safety pharmacology, or behavioral studies to access the effects of novel drugs on experimental animals. While providing rodents with opportunities for natural behaviors, and social interactions, these settings enhance the reliability and relevance of in-life phase findings in these domains. However, practical challenges emerge, especially in smaller animal facilities with limited resources, where strict environmental controls, such as those needed for infection studies, may conflict with the implementation of large cage spaces. Acknowledging these constraints is crucial for researchers to thoughtfully apply enrichment strategies aligned with experimental goals, ensuring ethical and effective utilization in diverse contexts.

#### Handling and training methods

**Fig. 2:**
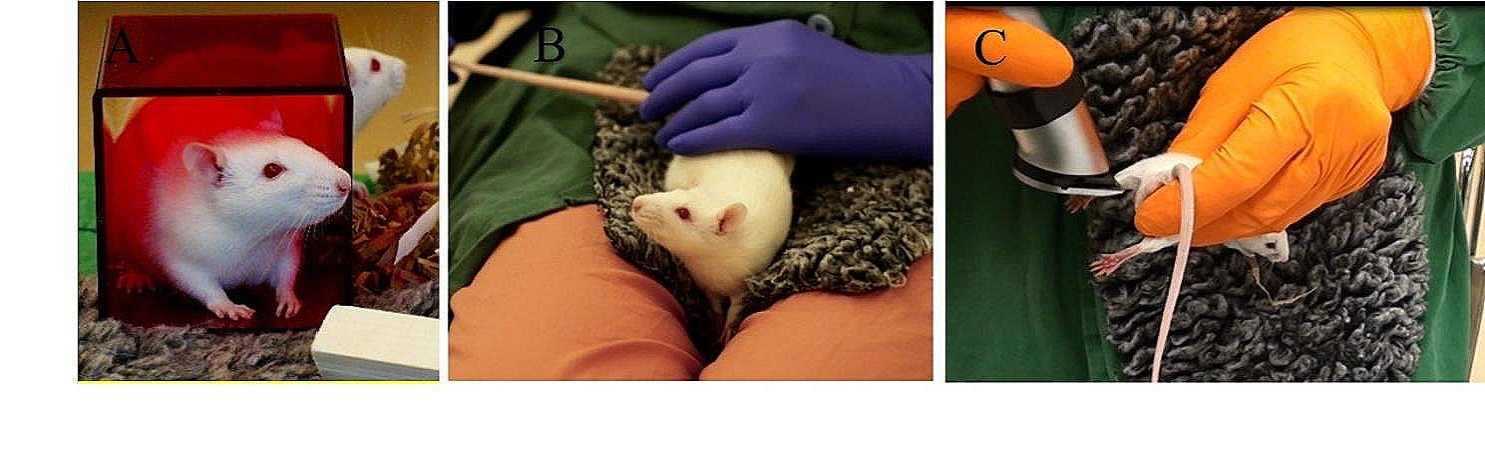
Handling and training methods of rodents according to the refinement principles. (**A**) Small tunnels, initially introduced as enriching elements within the home cage, serve a dual purpose by also becoming valuable tools for lifting the animals later. This method allows for gentle and non-invasive handling, as rodents naturally seek refuge in small, enclosed spaces. By incorporating these tunnels into their environment, researchers can establish a sense of familiarity and security, making it easier to handle the animals without causing undue stress. (**B**) A soft and comforting mat tailored for animals is depicted as another handling method. This mat not only provides a cozy resting surface but also facilitates gentle and stress-free animal handling. By using materials that are comfortable and familiar to the animals, researchers can create a positive association with handling procedures, reducing anxiety, and promoting cooperation. (**C**) Before saphenous vein sampling, familiarizing animals with technician grips and shaving machine sounds ensures well-trained and cooperative animals without the need for restraint. This training method involves exposing the animals to the sensory cues associated with the sampling procedure in a non-threatening manner. By gradually introducing these stimuli and pairing them with positive experiences, such as treats or rewards, researchers can desensitize the animals to potentially stressful aspects of the procedure, resulting in smoother and more reliable data collection while minimizing discomfort for the animals

Prioritizing non-restraint techniques for handling rodents holds paramount importance, as it effectively mitigates stress and anxiety during experimental procedures [25]. These approaches involve training rodents to willingly engage in procedures, sidestepping the need for physical restraint or forceful manipulation. Notably, tunnel handling has gained prominence in non-restraint methods; animals are gently guided into small tunnels (which can be provided in the home cage as an enrichment source and later can be used as a tool for handling), replacing tail grasping [25]. Sensini et al. (2020) found that mice favored exploring tunnels over interacting with the experimenter’s hands, displaying positive behaviors like touching and climbing while showing less defensive burrowing, highlighting a clear preference for tunnel exploration [26]. Henderson et al. (2020) demonstrated that mice handled through tunnel interactions exhibited heightened willingness to engage with handlers and decreased anxiety levels in behavioral tests even after repeated restraint, contrasting with those handled by their tails [27]. Also, use of a mouse lift, hand cup or other similar things to lift the animals from their home cage can also prove to be helpful. Conversely, tail handling, known to trigger fear and stress reactions, can negatively impact both experimental outcomes and animal welfare [28]. Therefore, adopting non-restraint methods not only respects animals’ autonomy but also curtails injury and potential discomfort during handling. Furthermore, incorporating specialized materials such as fleece bedding or plush mat which are designed for animals to rest on can help to establishe a soft and comfortable place for rodents during training and dosing procedures. This fleece bedding, indicative of animal’s own bedding, not only provides familiarity and comfort but also nurtures natural behaviors. Similarly, introducing fleecy autoclavable bedding in weighing boxes creates a calm and stress-free environment during weighing, simultaneously ensuring hygiene through autoclaving, aligning seamlessly with research standards [29]. Therefore, refined handling of rodents enhances their trust, reduces stress, and ensures cooperative behavior, crucial for accurate scientific observations and ethical treatment (Fig. 2).

Training animals before experimentation establishes trust and cooperation, ensuring that they are calm and willing to participate, leading to smoother procedures, reduced stress levels, and more reliable research outcomes. There are specific training methods for rodents that align with the principles of refinement, focusing on gentle handling, positive reinforcement, and reduced stress during procedures (Table 1).

**Table 1:** Various training methods for rodents in refinement efforts, highlighting their descriptions and corresponding research references

Training Method	Description	References
Positive Reinforcement	Utilizing positive reinforcement such as food rewards, enrichment materials, or social interactions establishes a favorable connection with the experimental environment, motivating rodents to engage willingly	[30]
Habituation and Desensitization	Gradual exposure to handling procedures and equipment to acclimate rodents to potentially stressful stimuli, reducing stress responses and improving compliance over time.	[31]
Enrichment-Based Training	Integrates enrichment activities into training sessions to provide mental stimulation and promote positive associations with handling, such as maze navigation for food rewards.	[32]
Virtual Reality Training	Utilizes VR simulations to familiarize rodents with handling procedures in a controlled environment, reducing stress responses and increasing cooperation during subsequent sessions.	[33]
Tailored Training Protocols	Customizes training plans to individual animal preferences and behavioral tendencies, optimizing outcomes by adapting to unique needs such as tactile or auditory preferences.	-

Training of the laboratory animals (approximately 4–5 times) upon their arrival can be divided into following different sessions which are critical for ensuring their involvement throughout the future experiments.

First training session: When laboratory animals first arrive at an animal facility, their initial training and handling plays a crucial role in their subsequent welfare. Scientific research provides valuable insights into the importance of structured training protocols during this critical period. An article by Swan et al. (2023) emphasizes the significance of gentle handling in the training of laboratory animals [34]. The article features the importance of general training and gentling protocols in contributing to the animals’ overall well-being and adaptability to the common laboratory procedures. Furthermore, a review by Mieske et al. (2022) highlights the impact of stimulating living environment using cognitive stimulation and mental training in laboratory rodents greatly benefits their welfare status [35]. Therefore, such type of training if given during the initial phase of acclimatization can greatly reduce the stress levels in experimental rodents.

In addition to these, understanding animal natural behaviors is crucial during the first training period. Animals fear novelty, odors, noise, sudden movements, pain, and a lack of control, exhibiting aversive behaviors such as freezing, fleeing, vocalizing, and altering body positions [36]. Recognizing varying fear levels in different animals can enhance the relevant observations during the training period. Early interactions should prioritize gentle stroking and playful handling, promoting the perception of friendly staff and their safe hands. Thus, the initial training session aims to foster trust and recognition, while observing individual stress reactivity and potential outliers. By incorporating gentle handling, mental stimulation, and understanding the animal’s natural fear response, researchers can promote the acclimatization and well-being of laboratory animals.

Subsequent training sessions: During these sessions, the primary focus shifts towards preparing animals for upcoming procedures. It’s essential to review the study protocol to identify specific body parts requiring extra handling and to introduce animals to necessary equipment. For instance, if oral dosing is planned, animals should be trained with the dose grip. By the last training session, a gentle introduction of water through a soft oral feeding tube is recommended. Training should encompass various sampling techniques as well. For example, if blood collection involves the tail vein, the tail should be stroked more often and gently pricked. In the case of saphenous vein sampling, animals should become accustomed to technician grips and the sound of the shaving machine. If restrainer tubes needed to be employed, such as for inhalation studies, animals should get familiarized with them before the first restraint procedure. The restrainer can be placed in the home cage of the animals for few days, to allow the animals to freely enter and explore the tube, so they get familiar with it. The goal of the final training session is for animals to exhibit calmness, relaxation, and curiosity.

Investing time in training rodents before commencing a study proves to be a time-saving measure. It prevents challenges during dosing and sampling procedures arising from stressed animals, offering instead a cooperative behavior. This familiarity enables technicians to accurately observe animal behavior during the study, differentiating between compound-induced responses and stress-related behaviors. Consequently, adopting refined handling and training methods is not only an objective to ensure welfare of laboratory rodents but also guarantees the production of dependable and credible research findings.

#### Dosing and sampling techniques

**Fig. 3:**
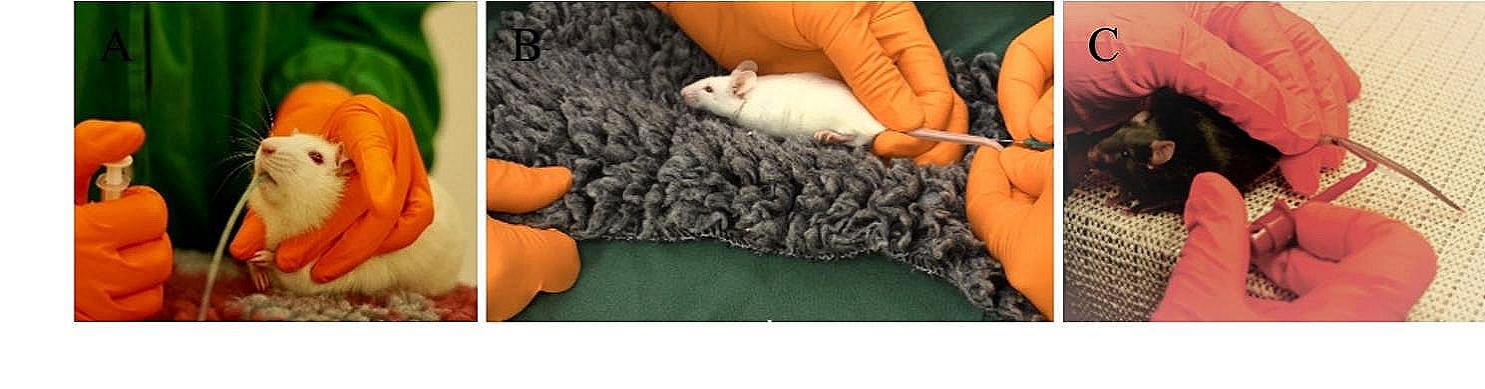
Dosing and sampling techniques for rodents. (**A**) Soft oral feeding tubes for rodents are depicted as a dosing technique aimed at reducing the risk of injury and discomfort during oral administration. These tubes are designed to be gentle on the animals’ mouths and throat, minimizing the likelihood of abrasions or irritation. By using soft materials and careful insertion techniques, researchers can ensure a more humane and stress-free experience for the animals, promoting their well-being while facilitating accurate dosing. (**B**) Intravenous or subcutaneous injections without physical restraint are shown as an alternative to traditional restraint methods. By administering injections without restraining the animals, researchers can reduce the stress and anxiety associated with physical immobilization. This approach allows the animals to remain more relaxed during the procedure, potentially minimizing discomfort and improving the overall quality of data collected. (**C**) Capillary micro-sampling is presented as a sampling technique that requires minimal blood volume, thereby reducing the impact on animal welfare while ensuring precise sampling. This method involves using specialized equipment to collect small blood samples from the rodents’ capillaries, typically from the tail vein. By minimizing the amount of blood drawn and utilizing precise sampling techniques, researchers can obtain the necessary data while minimizing the stress and discomfort experienced by the animals

Within the framework of the 3R-Refinement principles, rodent dosing and sampling methods play pivotal roles in minimizing the impact of experimental procedures on laboratory animals. A notable refinement in this regard involves the implementation of soft oral feeding tubes which prioritize animal comfort, reducing the likelihood of injury or discomfort during dosing [37]. This approach proves particularly advantageous when administering potentially irritant substances or repeated doses, ensuring a humane and stress-free experience [38]. Since the rodents are unable to vomit, therefore, using the smallest volume possible is recommended for the oral route of administration. Turner et al., 2012 found that orogastric gavage of aqueous solutions at 5 mL/kg does not negatively affect the welfare of laboratory rats acclimated to handling [39]. Additionally, intravenous, or subcutaneous injections, free from physical restraint, are crucial refinements that alleviate distress linked with traditional restraint methods [40]. Employing well-trained and cooperative animals enables injection administration without restraint, fostering a more comfortable and less aversive encounter for the rodents. The capillary micro-sampling technique is another noteworthy refinement for collecting small blood volumes, sufficient for the bioanalytic quantification in contrast to traditional large-volume methods [41]. This approach reduces animal suffering and hematoma formation, utilizing only a fraction of the conventional blood volume. Prioritizing less invasive methods, such as tail vein or saphenous vein sampling, over retro-orbital sampling, underscores ethical considerations [42]. Study from Meyer et al. (2020) showed that the blood sampling techniques from the vena facialis and retrobulbar sinus significantly affected mouse locomotor activity and anxiety-related behavior, while tail vessel bleeding had minimal impact on assessed physiological and behavioral parameters [43]. Minimally invasive techniques not only uphold rodent welfare but also mitigate infection or complications tied to more invasive methods, ultimately diminishing physiological stress responses for more accurate and reliable data [44]. Furthermore, animals experiencing reduced stress during dosing and sampling are likely to exhibit heightened cooperation in subsequent procedures, fostering a favorable research atmosphere (Fig. 3).

#### Assessment and management of pain

Effective pain assessment and management stands as a basis for mitigating animals’ suffering and safeguarding their welfare throughout the research journey. Two commonly observed behaviors in many rodent strains are digging and nest building. However, when rats are experiencing neuropathic or inflammatory pain, research has demonstrated a decrease in their digging behavior [45]. This change in behavior can be easily evaluated during experimental conditions. Employing suitable analgesics, researchers can soften discomfort arising from experimental manipulations, surgical interventions, or disease models, thereby advocating a more compassionate and humane approach to animal research [46, 47]. Gaseous anesthesia’s quick induction and recovery, along with its non-invasive administration advantages, are especially beneficial for minor procedures; however, for more complex and invasive surgeries, the inclusion of injectable analgesics becomes crucial [48]. Remarkably, micro-dosing of intravenous (*i.v.*) analgesics, as contrast to extensive intraperitoneal (*i.p.*) injections, unveils notable advantages. Primarily, *i.v.* micro-dosing facilitates precise, targeted analgesic administration, underpinning an accurate and effective pain management regimen. This strategy assures animals receive the requisite pain relief dose, mitigating the risk of suboptimal or excessive dosing. Secondly, *i.v.* injections, being less invasive than *i.p.* counterparts, cause minimal discomfort, excluding the substantial fluid volume associated with *i.p.* injections, which can at times puncture internal organs.

Researchers should be encouraged to explore alternative pain management strategies, such as localized anesthesia or multimodal analgesia—an approach entailing the combination of diverse analgesic agents for more comprehensive pain alleviation. This strategy holds promise in potentially diminishing individual analgesic dosages, curbing side effects, and enhancing overall pain management outcomes. Besides, it is necessary to employ non-invasive pain assessment techniques for gauging analgesia efficacy and monitoring animals’ pain status [49]. Regular evaluation of behavioral and physiological pain cues, encompassing alterations in activity, posture, vocalization, and vital signs, aids in discerning the level of pain experienced by animals, facilitating adjustments to analgesic protocols as necessary [50].

#### Animal welfare monitoring

Regular and systematic monitoring of animal welfare is essential to identify any signs of distress, pain, or discomfort, enabling researchers to promptly intervene and provide appropriate care. Various welfare indicators, both behavioral and physiological, should be utilized to assess the animals’ well-being. Behavioral indicators may include changes in activity levels, posture, grooming, social interactions, and exploratory behaviors [51]. Physiological indicators, such as body weight, temperature, heart rate, and hormone levels, should also be monitored to provide insights into the animals’ overall health and stress responses. Furthermore, researchers should be encouraged to employ refined monitoring techniques that minimize the potential disturbance to the animals [52]. Non-invasive methods, such as remote monitoring and video recording, allow researchers to observe animals in their natural environment without direct human interference, thus reducing potential stress-related biases in the assessment [53]. Regular training of research personnel in animal welfare assessment is vital to recognize subtle changes in animal behavior and physical condition, enabling early detection of welfare issues and timely intervention.

#### Establishment of humane endpoints

Establishing humane endpoints within experimental protocols is a pivotal practice aimed at mitigating extreme distress and pain experienced by laboratory animals during studies [54]. These endpoints outline the thresholds beyond which animals should not be subjected to further procedures, ensuring that their welfare and well-being are paramount. By closely monitoring animal behavior, physiological parameters, and overall health, researchers and veterinarians can proactively identify signs of significant distress or suffering. For instance, David et al. (2000) demonstrated a systemic approach using numerical rating, simple description, scoring clinical signs such as lethargy or severe weight loss, served as reliable indicators for reaching humane endpoints in rodent models of disease [55]. This approach utilized clinical signs to gauge the extent to which an animal’s physical and mental condition has deviated from the normal, using the severity of these deviations as an assessment criterion. When these predetermined endpoints are reached, appropriate actions must be taken, such as terminating the experiment or implementing immediate interventions to alleviate the animals’ discomfort [56]. This approach not only aligns with ethical considerations but also underscores the responsibility of researchers to safeguard animal welfare.

#### Euthanasia procedures

At the conclusion of experiments, the implementation of humane methods for rodent euthanasia is of paramount importance to uphold ethical standards for experiments on laboratory animals [57]. Euthanasia procedures should be swift and minimize pain and distress, aligning with the principles of 3R-Refinement. Deciding how to euthanize laboratory animals is complex and should involve consultation with a specialized veterinarian. Clarkson et al. (2022) highlighted that the physical methods like cervical dislocation, decapitation, and concussion raise welfare concerns due to potential inaccuracies and high failure rates therefore quality-controlled training programs and specialized tools are needed to improve success rates and reliability [58]. To ensure the chosen method is suitable for the research animals and the study’s goals, conducting a ‘preliminary study’ is a good way to determine the most appropriate euthanasia method for the specific study and groups of rodents [7]. Following euthanasia, proper disposal of carcasses should be carried out with consideration for biosecurity and environmental impact. Adhering to these humane protocols for euthanasia is not only a moral obligation but also underscores the dedication to maintaining the highest standards of animal care and welfare throughout the research process.

#### Collaboration with animal care and ethics committees

Animal Care and Ethics Committees play a crucial role in reviewing and approving research protocols involving animal experimentation, ensuring that all experiments meet the highest ethical standards and comply with relevant regulations [59]. Collaboration with these committees not only ensures compliance with ethical guidelines but also enhances the quality and validity of research outcomes. Animal Ethics Committees also facilitate training and education initiatives for researchers to promote awareness and understanding of the 3R-Refinement principles. The 3Rs Center of Europe, for example, holds a significant role in fortifying the 3R-Refinement principles within Europe’s scientific landscape [60]. Through collaboration with researchers, institutions, and regulatory bodies, these centers facilitate the dissemination of best practices, knowledge sharing, and the development of innovative methods that prioritize animal welfare. Therefore, by engaging with animal care committees, researchers can ensure that their experiments adhere to ethical standards, prioritize animal welfare, and contribute to the advancement of science while minimizing animal suffering.

The summarized table below (Table 2) encapsulates key components of the 3R-Refinement principles, offering a comprehensive overview of ethical practices in laboratory animal research, ranging from animal arrival and acclimatization to euthanasia procedures.

**Table 2:** Summary of key components of the 3R-Refinement principles discussed in the paper

Key Component	Summary	References
Arrival and Acclimatization of Animals	Proper unpacking protocols, including disinfection and health monitoring, ensure animal welfare upon arrival. Gradual acclimatization to new environments and gentle handling techniques, reduce stress and foster positive relationships with caretakers.	[10–15]
Housing and Enrichment Environment	Enriched housing, including large cage spaces and social groupings, promotes natural behaviors and reduces stress. Incorporating various enrichment materials and observation boxes creates stimulating living conditions while facilitating research on animal behavior.	[16–24]
Handling and Training Methods	Non-restraint handling techniques, such as tunnel handling, promote animal welfare and cooperation during experimental procedures. Gentle handling and the use of soft bedding materials establish trust and comfort, enhancing scientific observations and ethical treatment.	[25–36]
Dosing and Sampling Techniques	Refinement in dosing and sampling methods, such as soft oral feeding tubes and non-restraint injections, prioritizes animal comfort and minimizes distress. Capillary micro-sampling techniques reduce blood volume requirements and uphold ethical considerations.	[37–44]
Assessment and Management of Pain	Effective pain assessment and management strategies, including the use of analgesics and non-invasive monitoring techniques, mitigate animal suffering during experimental procedures. Establishing humane endpoints and employing swift euthanasia procedures further uphold ethical standards.	[45–50]
Animal Welfare Monitoring	Regular monitoring of animal welfare indicators, both behavioral and physiological, allows for early detection of distress and timely intervention.	[51–53]
Establishment of humane endpoints	Monitoring animal behavior and physiological parameters. Setting thresholds to avoid extreme distress or suffering, and taking appropriate actions when endpoints are reached.	[54–56]
Euthanasia Procedures	Swift and humane methods for euthanasia. Consultation with specialized veterinarians for method selection along with design of preliminary studies to determine appropriate euthanasia methods. Consideration of biosecurity and environmental impact in carcass disposal.	[57, 58, 7]
Collaboration with Animal Care and Ethics Committees	Review and approval of research protocols so that they follow ethical standards and regulations. Facilitation of training and education initiatives. Dissemination of best practices and knowledge sharing. Contribution to the advancement of ethical animal research practices.	[59, 60]

### Impact and benefits of 3R-refinement principles

The 3R-Refinement protocol has a profound impact on laboratory animal research, leading to numerous benefits for both animals and researchers. The impact and benefits of the 3R-Refinement protocol as discussed below underscore the critical role of ethical considerations in laboratory animal research, promoting a friendly relationship between scientific advancement and animal welfare.

#### Enhanced animal welfare and well-being

Treating laboratory animals as valuable colleagues, rather than mere research subjects, fosters a positive and respectful approach towards their care and handling. By adopting refined methodologies and practices, researchers prioritize the humane treatment of animals, reducing potential distress and suffering during experimental procedures. This approach leads to improved psychological welfare, as evidenced by reduced stress and anxiety levels in laboratory animals [61]. The 3R-Refinement principles not only align with ethical considerations but also positively impact the emotional and physiological welfare of laboratory animals, ultimately fostering a more compassionate and responsible approach to animal research.

#### Improved scientific validity and reliability

The implementation of the 3R-Refinement protocol brings about substantial benefits to the scientific community, particularly in terms of improved scientific validity and reliability in laboratory animal research. By adhering to these principles researchers can refine experimental methodologies, leading to a reduction in potential confounding factors and improved data accuracy [62]. The adoption of refined techniques and non-invasive procedures minimizes stress-related responses in animals, thereby provides a more accurate representation of the true biological effects being studied [63]. This not only contribute to better reproducibility and consistency of research outcomes but also increases the overall robustness of scientific findings. Moreover, the 3R-Refinement principles encourage the use of appropriate control groups, randomization, and blinding, further enhancing the internal validity of experimental designs. By controlling potential bias and minimizing sources of inconsistency, researchers can achieve more reliable and precise results.

#### Reduction in variability and animal numbers

The implementation of the 3R-Refinement protocol demonstrates significant impact and benefits on reducing variability and animal numbers in laboratory animal research. By adopting refined methodologies and techniques, researchers obtain more precise and reliable data with fewer animals. By incorporating refined experimental designs, researchers minimize sources of variability, leading to more consistent and reproducible results [64]. Furthermore, this also encourage researchers to admire the 3R-Reduction principle i.e., carefully consider the statistical power of their experiments and use the minimum number of animals necessary to achieve meaningful and statistically significant results. This approach not only reduce the overall use of animals in research but also improve the efficiency and cost-effectiveness of scientific studies [65]. The 3R-Refinement protocol also promotes the sharing of best practices and standardization of methodologies, further contributing to a reduction in experimental variability across different research groups.

#### Positive feedback from researchers and animal care staff

The implementation of the 3R-Refinement Protocol in laboratory animal research has a profound impact on eliciting positive feedback from researchers and animal care staff. By prioritizing animal welfare and employing refined methodologies, the protocol fosters a culture of empathy and dedication to ethical practices among researchers [66]. Researchers appreciate the opportunity to work with humane and compassionate approaches, which not only align with their ethical values but also contribute to the generation of reliable and meaningful research outcomes. Animal care staff also experience a sense of fulfillment, knowing that they are actively contributing to the well-being and comfort of the animals under their care. The positive feedback from both researchers and animal care staff reflects a shared commitment to prioritizing animal welfare while advancing scientific knowledge.

### Promoting the adoption of 3R-refinement principles

Adoption of the 3R-Refinement Protocol is integral to fostering a culture of ethical and compassionate research practices. This initiative aims to elevate animal welfare while enhancing the scientific validity of experimental outcomes and can be achieved through various proactive measures as discussed below.

#### Collaboration within research institutes and stakeholders

By actively engaging with and sharing experiences, knowledge, and best practices, research institutions can collectively work towards implementing the 3R-Refinement principles more effectively. Collaboration fosters the exchange of innovative ideas and solutions through creation of common databases, alternative literature searches etc. which further facilitates the development of refined methodologies that enhance animal welfare and reduce variability in research outcomes [67]. These collaborations promote a harmonized approach to ethical animal research practices, ensuring improved animal welfare, enhanced scientific validity, and the promotion of socially responsible research practices.

#### Broadcasting best practices and guidelines

Propagation or spread of best practices and guidelines plays a crucial role in achieving widespread adoption among research institutions and stakeholders. By sharing successful implementations of the 3R-Refinement principles, researchers can inspire others to follow suit, fostering a culture of compassion and care for laboratory animals. Peer-reviewed publications serve as platforms for researchers to present their refined methodologies, enriched housing environments, and effective pain management strategies, resulting in improved animal welfare and scientifically robust research outcomes [68]. Furthermore, the dissemination of best practices extends beyond academic publications, with online resources and databases providing a repository of successful case studies and refined techniques [67].

#### Training programs and workshops

Promoting the adoption of the 3R-Refinement protocol is a multifaceted endeavor, and training programs and workshops play a pivotal role in achieving this goal. These educational initiatives provide a platform for researchers, animal care staff, and ethics committee members to learn about the Refinement principles and their practical implementation in laboratory animal research [69]. Training programs and workshops offer a space for open discussions, knowledge sharing, and the exchange of experiences, developing a collaborative and informed approach to animal research [70]. By engaging experts in the field as facilitators, these programs ensure that participants receive up-to-date information and evidence-based guidelines. Besides, these initiatives encourage researchers to critically evaluate their experimental designs and explore innovative ways to reduce animal sufferings while maintaining scientific rigor.

#### Engagement of regulatory bodies and ethical review committees

Implementation of the 3R-Refinement protocol is greatly facilitated by the active involvement of regulatory bodies and ethical review committees. These organizations play a critical role in shaping the ethical landscape of laboratory animal research by developing and implementing guidelines and policies that align with the 3R-Refinement principles [71]. Their scrutiny encourages researchers to critically assess their experimental designs, consider refinement approaches, and justify the number of animals used, ultimately fostering a culture of ethical and responsible animal research [72]. Additionally, regulatory bodies and ethical review committees can collaborate with researchers and animal technicians to develop educational resources and training programs that provide guidance on the practical implementation of the 3R-Refinement principles. Furthermore, the feedback and recommendations provided by these bodies facilitate continuous improvement and refinement of research protocols, promoting a collective commitment to animal welfare and pursuit of scientific excellence.

## Conclusions

The comprehensive framework presented in this review features the significance of considering animal welfare at every stage of research, the influence of such practices on animals and scientific community and possible ways of its implementation. By embracing the 3R-Refinement Protocol, researchers not only uphold the highest ethical standards but also advance the scientific quality and validity of their work. This approach fosters a culture of empathy, responsibility, and collaboration among researchers, animal care staff, and regulatory bodies. By embracing the refinement principles, researchers pave the way for a future where innovative science and ethical considerations coexist, benefitting both humanity and the animals.

## Data Availability

Not applicable.

## Abbreviations

*i.v.*: intravenous
*i.p.*: intraperitoneal
